# Green and Highly Efficient Synthesis of 2-Arylbenzothiazoles Using Glycerol without Catalyst at Ambient Temperature

**DOI:** 10.3390/molecules17056011

**Published:** 2012-05-18

**Authors:** Kamal Usef Sadek, Ramadan Ahmed Mekheimer, Afaf Mohamed Abdel Hameed, Fatma Elnahas, Mohamed Hilmy Elnagdi

**Affiliations:** 1 Chemistry Department, Faculty of Science, El-Minia University, El-Minia 61519, Egypt; 2 Chemistry Department, Faculty of Science for Girls, King Abdulaziz University, P.O. Box 50918, Jeddah 21533, Saudi Arabia; 3 Chemistry Department, Faculty of Science, Kuwait University, P.O. Box 5969, Safat 13060, Kuwait

**Keywords:** synthesis, 2-arylbenzothiazoles, glycerol, 2-aminothiophenols

## Abstract

A one-pot and clean synthesis of 2-arylbenzothiazoles via the ambient temperature reaction of 2-aminothiophenols and aromatic aldehydes without catalyst in glycerol as a green solvent has been reported.

## 1. Introduction

With technological and scientific advancement comes cost, and with respect to this issue, the most important cost to be dealt with is the impact of these technologies on our environment [1]. Most organic reactions occur in a liquid phase. Taking into account the impact of chemical processes on the environment, the search for green solvents has become a great challenge in organic synthesis, as solvents are responsible for a large part of the waste and pollution generated by chemical processes, a key factor in any green chemical process is solvent selection [2].

Glycerol is a non-toxic, biodegradable and recyclable liquid that is highly inert, stable and also dissolves organic compounds that are poorly miscible in water. It combines the advantages of water (low toxicity, low price, wide availability) and ionic liquids (highly boiling point, low vapor pressure) [3]. These advantages make it ideal for use as a sustainable solvent in organic synthesis.

2-Arylbenzothiazoles can be found in a variety of natural products as well as a number of biologically active compounds [4]. They are a class of potent and selective antitumor agents which exhibit nanomolar inhibitory activity against a range of human breast, colon and renal cell lines *in vitro* [5]. In addition, they possess significant utility as imaging agents for *β*-amyloid, antitubercullotic, chemiluminescent agents, calcium channel antagonists, antiparasitics and photosensitizers [6,7,8,9,10].

To date, two common strategies have been developed used for the synthesis of 2-arylbenzothiazoles. The first approach is through arylation of benzothiazole with aryl bromides catalyzed by Pd(OAc)_2_, Cs_2_CO_3_ and CuBr with P(*t*-Bu)_3_ as a ligand at 150 °C in a sealed tube [11], or Suzuki biaryl coupling of 2-bromothiazoles with arylboronic acids [12]. The second method is via condensation of 2-aminothiophenols with substituted nitriles, carboxylic acids, aryl chlorides, esters or aldehydes [13]. A number of catalysts such as (PmIm)Br [14], I_2_ [15], ZrOCl_2_•8H_2_O [16], TMSCl [17], PCC [18], CAN [19], molecular oxygen [20], H_2_O_2_ [21], MnO_2_ [22], Baker’s yeast [23], animal bone meal [24], electrooxidation [25], Na_2_S_2_O_5_ in refluxing DMF [26] have been used to perform the cyclization step. Recently, some green approaches for the preparation of 2-arylbenzothiazoles based on the direct condensation of 2-aminothiophenols with aromatic aldehydes have been reported. They involve the use of PTSA either in water at 70 °C or PEG, 200/400 under microwave heating [27,28], Cu(OAc)_2_/MCM41 supported catalyst under ultrasound irradiation [29], NIBTS at ambient temperature under solvent free conditions [30], Dowex 50W reusable catalyst in water at 70 °C [31], TCCA in MeTHF at ambient temperature [32], heating in water at 110 °C [33], 2,4,6-trichloro-1,3,5-triazine under mild conditions [34], H_2_SO_4_/SiO_2_ as a reusable catalyst at room temperature [35] or sulfamic acid as a reusable catalyst in water at room temperature [36]. Although, these methods each have specific merits, they suffering from some drawbacks such as high temperature conditions, long reaction times and sometimes low yields. In addition, the catalysts employed in most of these cyclization steps are not always inexpensive or eco-friendly, and consequently, serious environmental pollution often results when the catalysts contaminate the environment. Since glycerol is able to dissolve organic compounds that are poorly miscible in water, its use as a green solvent without catalyst would overcome such drawbacks. Also, conducting the reaction at ambient temperature will increase the energy efficiency of the reaction. Very recently, a one-step synthesis of 2-arylbenzothiazoles from the reaction of *gem*-dibromomethylarenes with 2-aminothiophenol employing *t*-BuOK and a catalytic amount of iodine in pyridine at reflux temperature has been reported [37]. In 2012, Cheng and co-workers have described another approach to 2-arylbenzothiazoles via Jacobson’s cyclization of thiobenzanilides catalyzed by aerobic visible light, but this route requires a multistep reaction sequence [38]. In this regard, employing a benign solvent under catalyst free conditions for the synthesis of the target molecules will be of great interest in the area of green chemistry.

In continuation of our work aiming at development of efficient, simple and green technologies for the synthesis of heterocycles [39,40,41], we reported herein a one-pot synthesis of 2-arylbenzothiazoles from the ambient temperature reaction of 2-aminothiophenols **1** with aromatic aldehydes **2** in glycerol under catalyst free conditions.

## 2. Results and Discussion

**Scheme 1:**
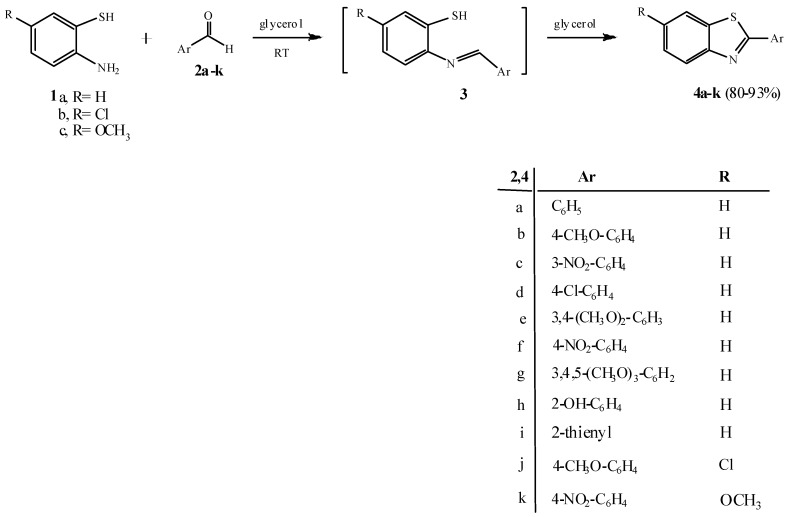
One-pot synthesis of 2-arylbenzothiazoles **4a–k**.

With the initial aim of optimising the experimental conditions, we explored the reaction of 2-aminothiophenol (**1a**) and benzaldehyde (**2a**) using glycerol as a solvent to afford the desired 2-phenyl-1,3-benzothiazole (**4a**) (Scheme 1). Thus, when a mixture of **1a** (0.1 mol) and **2a** (0.1 mol) in glycerol (10 mL) was stirred at room temperature, it was observed that reactants were recovered almost unchanged after a long period of time. The reaction promoted by just heating the reaction mixture until the reactants were dissolved and then left at room temperature for 30 min afforded **4a** in excellent yield. The structure of compound **4a** could be established on the basis of analytical and spectral data and comparison with authentic specimen prepared via an alternative route [18]. Similarly, compounds **1a–c** reacted with **2b–k**, under the same reaction conditions, to afford **4b–k** in excellent yields.

**Scheme 2:**
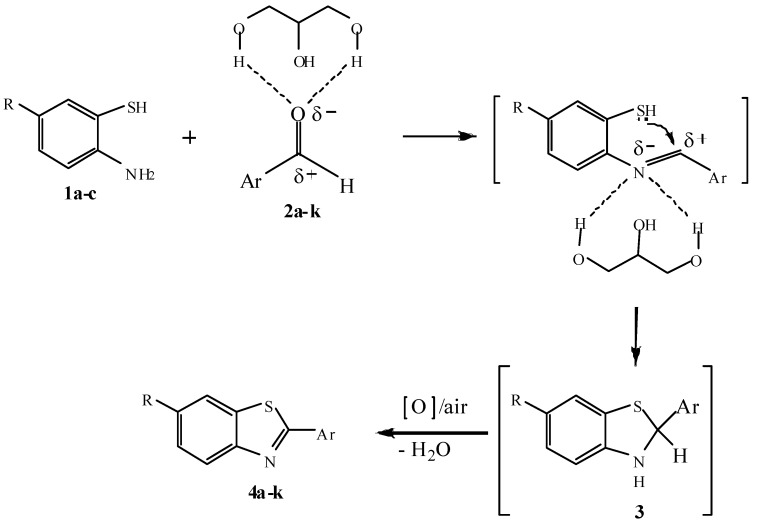
A proposed mechanism for the formation of 2-arylbenzothiazoles **4a–k**.

In order to study the effect of solvent on the rate of the reaction we carried out the same reaction in different solvents, such as H_2_O, acetone and CHCl_3_, under the same reaction conditions, and the reactants were recovered almost unchanged. Next, we studied the effect of aromatic aldehyde substituents on the reaction rate and the overall yield. With both electron withdrawing and electron donating groups, the reaction proceeds smoothly, with a slight increase in the yield when the aryl substituent bore an electron withdrawing group. We also studied the generality and applicability of such protocol via examining the reaction of substituted 2-aminothiophenols bearing both electron donating and electron withdrawing groups. In both cases, the reaction proceeds smoothly with good yields. Although until now the reasons behind the promoting effect of glycerol are still unclear, a mechanism to account for the formation of **4a–k** is postulated in Scheme 2.

The highly solving action of glycerol on the reactants makes them readily available to interact and the carbonyl carbon of the aldehyde will be activated because of the intermolecular hydrogen bonding with the hydroxyl groups of glycerol. In addition, the formed intermediates could be stabilized by several types of complexations and hydrogen bonding with the hydroxyl groups of glycerol. This was followed by cyclization to form the corresponding thiazoline intermediate **3** which, on dehydrogenation, via aerial oxidation, is converted to the thiazole **4** which will enjoy the aromaticity (*i.e*., stabilization) of the benzothiazole ring system. Conducting the reaction of **1a** with **2a** in the absence of air resulted in a sluggish reaction inconvenient for complete product formation even after a longer reaction time. It is worth mentioning that strong oxidants or more interestingly catalytic aerobic oxidation involving oxygen as a terminal oxidant have received considerable attention in the synthesis of benzothiazoles [21]. The advantage of our protocol is the use of only atmospheric air as the oxidant at ambient temperature. However, we cannot totally rule out that the presence of trace amount of acidic and basic derivatives in glycerol can act as possible catalysts.

## 3. Experimental

### 3.1. General

Melting points were determined on a Gallenkamp melting point apparatus and are uncorrected. 2-Aminothiophenol (**1a–c**) and aldehydes **2a–k** were commercially available. Infrared spectra were measured with a Schimadzu Model 470 spectrophotometer. The ^1^H- and ^13^C-NMR spectra were recorded on a Bruker AM 400 spectrometer (at 400 MHz for ^1^H-NMR and 100 MHz for ^13^C-NMR) with DMSO-*d*_6_ as solvent and TMS as internal reference, chemical shifts are expressed as δ ppm. Mass spectra were measured on a GCMS-QP1000EX mass spectrometer. Analytical data were determined on the Microanalytical Data Unit at Kuwait University. Analytical TLC was performed with a silica gel plates using silica gel 60 PF_254_ (Merck).

### 3.2. Synthesis of 2-Arylbenzothiazoles ***4a–k***

A mixture of thiophenols **1a–c** (1.25 g, 10 mmol) and the appropriate aldehyde **2** (10 mmol) in glycerol (10 mL) was heated until a clear solution was obtained and then left at room temperature for 0.5–5 h (TLC control). The reaction mixture was quenched with water and the resulting solid product was collected by filtration, dried and recrystallised from EtOH to afford compounds **4a–k**.

*2-Phenyl-1,3-benzothiazole* (**4a**). Yield: 92%; reaction time 0.5 h; mp 112–113 °C (Lit. [18] 115–116); IR (KBr): *ν* = 3,060, 3,020, 1,609, 1,590 cm^−1^; ^1^H-NMR: δ = 8.17 (d, 1H), 8.12–8.07 (m, 3H), 7.59–7.50 (m, 4H), 7.45 (t, 1H) ppm; ^13^C-NMR: δ = 167.30, 153.56, 134.45, 132.85, 131.44, 129.42, 127.20, 126.68, 125.57, 122.90, 122.38 ppm; MS *m*/*z* (rel. int. %) 211 (M^+^, 100); Anal. Calcd. for C_13_H_9_NS (211.28): C, 73.90; H, 4.29; N, 6.63; S, 15.18. Found: C, 73.79; H, 4.19; N, 6.81; S, 14.98.

*2-(4-Methoxyphenyl)-1,3-benzothiazole* (**4b**). Yield 90%; reaction time 4h; mp 126–128 °C (Lit. [25] 119–120 °C); IR (KBr): *ν* = 3,100, 3,060, 1,605, 1,585 cm^−1^; ^1^H-NMR: δ = 8.12 (d, 1H), 8.1–8.0 (m, 3H), 7.53 (t, 1H), 7.45 (t, 1H), 7.13 (d, 2H), 3.85 (s, 3H) ppm; ^13^C-NMR: δ = 167.05, 153.67, 134.23, 128.88, 126.52, 125.52, 125.11, 122.47, 122.20, 114.75, 55.5 ppm; MS *m*/*z* (rel. int. %) 241 (M^+^, 100); Anal. Calcd. for C_14_H_11_NOS (241.31): C, 69.68; H, 4.59; N, 5.80; S, 13.29. Found: C, 69.55; H, 4.52; N, 5.94; S, 13.18.

*2-(3-Nitrophenyl)-1,3-benzothiazole* (**4c**). Yield 92%; reaction time 2 h; mp 184–186 °C (Lit. [18] 181–183 °C); IR (KBr): *ν* = 3,080, 3,035, 1,612, 1,580 cm^−1^; ^1^H-NMR: δ = 8.84 (s, 1H), 8.44 (d, 1H), 8.41 (d, 1H), 8.24 (d, 1H), 8.16 (d, 1H), 7.88 (t, 1H), 7.60 (t, 1H), 7.54 (t, 1H) ppm; ^13^C-NMR: δ = 161.9, 157.5, 149.4, 142.8, 133.3, 130.7, 130.2, 126.5, 126.3, 122.5, 120.3, 119.5, 111.8 ppm; MS *m*/*z* (rel. int. %) 256.0 (M^+^, 100); Anal. Calcd. for C_13_H_8_N_2_O_2_S (256.28): C, 60.93; H, 3.15; N, 10.93; S, 12.51. Found: C, 60.86; H, 3.27; N, 11.02; S, 12.64.

*2-(4-Chlorophenyl)-1,3-benzothiazole* (**4d**). Yield 93%; reaction time 3 h; mp 116–118 °C (Lit. [25] 115–117 °C); IR (KBr): *ν* = 3,088, 3,030, 1,615, 1,590 cm^−1^; ^1^H-NMR: δ = 8.18 (d, 1H), 8.09 (d, 1H), 7.68 (d, 2H), 7.57 (d, 2H), 7.48 (m, 2H) ppm; Anal. Calcd. for C_13_H_8_ClNS (245.73): C, 63.54; H, 3.28; Cl, 14.43; N, 5.70; S, 13.05. Found: C, 63.47; H, 3.39; Cl, 14.58; N, 5.76; S, 12.87.

*2-(3,4-Dimethoxyphenyl)-1,3-benzothiazole* (**4e**). Yield 83%; reaction time 4 h; mp 130–132 °C (Lit. [19] 130–132 °C); IR (KBr): *ν* = 3,078, 3,052, 2,962, 2,835, 1,600 cm^−1^; ^1^H-NMR: δ = 8.10 (d, 1H), 8.09–8.02 (m, 1H), 7.66–7.60 (m, 2H), 7.53 (t, 1H), 7.41 (t, 1H), 7.12 (d, 1H), 3.88 (s, 3H), 3.85 (s, 3H) ppm; MS *m*/*z* (rel. int. %) 271 (M^+^, 100); Anal. Calcd. for C_15_H_13_NO_2_S (271.33): C, 66.40; H, 4.83; N, 5.16; S, 11.82. Found: C, 66.36; H, 4.73; N, 5.25; S, 11.69.

*2-(4-Nitrophenyl)-1,3-benzothiazole* (**4f**). Yield 94%; reaction time 2 h; mp 224–225 °C (Lit. [25] 224–226 °C); IR (KBr): *ν* = 3,082, 3,035, 1,615, 1,580 cm^−1^; ^1^H-NMR: δ = 8.32 (d, 2H), 8.21 (d, 1H), 8.10 (d, 2H), 8.01 (d, 1H), 7.53–7.44 (m, 2H) ppm; MS *m*/*z* (rel. int. %) 256.0 (M^+^, 100); Anal. Calcd. for C_13_H_8_N_2_O_2_S (256.28): C, 60.93; H, 3.15; N, 10.93; S, 12.51. Found: C, 60.88; H, 3.25; N, 11.05; S, 12.64.

*2-(3,4,5-Timethoxyphenyl)-1,3-benzothiazole* (**4g**). Yield 82%; reaction time 5 h; mp 130–131 °C; IR (KBr): *ν* = 3,082, 3,055, 2,965, 2,833, 1,600 cm^−1^; ^1^H-NMR: δ = 8.09–8.02 (m, 1H), 7.66–7.60 (m, 2H), 7.53 (t, 1H), 7.41 (s, 1H), 7.22 (s, 1H), 3.89 (s, 3H), 3.87 (s, 3H), 3.84 (s, 3H) ppm; Anal. Calcd. for C_16_H_15_NO_3_S (301.36): C, 63.77; H, 5.02; N, 4.65; S, 10.64. Found: C, 63.66; H, 5.03; N, 4.56; S, 10.72.

*2-(2-Hydroxyphenyl)-1,3-benzothiazole* (**4h**). Yield 80%; reaction time 5 h; mp 123–124 °C; IR (KBr): *ν* = 3,450, 3,080, 3,042, 1,625 cm^−1^; ^1^H-NMR: δ = 8.21 (d, 1H), 8.16–8.01 (m, 3H), 7.62 (t, 1H), 7.53 (t, 1H), 7.07 (d, 2H) ppm; Anal. Calcd. for C_13_H_9_NOS (227.28): C, 68.70; H, 3.99; N, 6.16; S, 14.11. Found: C, 68.73; H, 4.01; N, 6.21; S, 14.24.

*2-Thienyl-1,3-benzothiazole* (**4i**). Yield 81%; reaction time 5 h; mp 97–99 °C (Lit. [19] 97–99 °C); IR (KBr): *ν* = 3,083, 3,040, 1,630 cm^−1^; ^1^H-NMR: δ = 8.22 (d, 1H), 8.14 (d, 1H), 7.72 (d, 1H), 7.69 (d, 1H), 7.63–7.54 (m, 2H), 7.31 (t, 1H) ppm; MS *m*/*z* (rel. int. %) 217.0 (M^+^, 100); Anal. Calcd. for C_11_H_7_NS_2_ (217.31): C, 60.80; H, 3.25; N, 6.45; S, 29.51. Found: C, 60.71; H, 3.19; N, 6.62; S, 29.33.

*6-Chloro-2-(4-methoxyphenyl)benzothiazole* (**4j**). Yield 88%; reaction time 4 h; mp 137–138 °C (Lit. [28] 136–138 °C); IR (KBr): *ν* = 3,090, 3,060, 1,610, 1,590 cm^−1^; ^1^H-NMR: δ = 8.19 (s, 1H), 8.02 (d, 2H), 7.89 (d, 1H), 7.68 (d, 1H), 7.08 (d, 2H), 3.83 (s, 3H) ppm; Anal. Calcd. for C_14_H_10_ClNOS (275.75): C, 60.98; H, 3.66; N, 5.08. Found: C, 60.88; H, 3.96; N, 5.22.

*6-Methoxy-2-(4-nitrophenyl)benzothiazole* (**4k**). Yield 80%; reaction time 5 h; mp 156–157 °C (lit. [28] 156–158 °C); IR (KBr): *ν* = 3,080, 3,035, 1,620, 1,580 cm^−1^; ^1^H-NMR: δ = 8.23 (d, 2H), 8.08 (d, 2H), 7.73 (s, 1H), 7.58 (d, 1H), 7.19 (d, 1H), 3.83 (s, 3H) ppm; ^13^C-NMR: δ = 170.0, 156.7, 149.6, 147.9, 146.7, 136.8, 128.4, 124.6, 122.8, 114.7, 104.9, 55.8 ppm; MS *m*/*z* (rel. int. %) 286 (M^+^, 100); Anal. Calcd. for C_14_H_10_N_2_O_3_S (286.31): C, 58.73; H, 3.52; N, 9.78 . Found: C, 58.66; H, 3.55; N, 9.66.

## 4. Conclusions

Glycerol has been employed for the first time as a benign solvent for the one-pot synthesis of 2-arylbenzothiazoles under catalyst free conditions and at ambient temperature. The procedure proved to be simple both in conducting the reaction and isolation of the products. The yields obtained are excellent and the products were recovered in pure form from the reaction mixture. To the best of our knowledge, it is one of the few reported one-pot syntheses of 2-arylbenzothiazoles, from the reaction of 2-aminothiophenol with aromatic aldehydes, at ambient temperature without catalyst.
